# Should the first degree relatives of type 2 diabetic patients with isolated impaired fasting glucose be considered for a diabetes primary prevention program?

**Published:** 2010

**Authors:** Bijan Iraj, Nader Taheri, Massoud Amini, Payvand Amini, Ashraf Aminorroaya

**Affiliations:** a Assistant Professor of Endocrinology, Isfahan Endocrine and Metabolism Research Center, Isfahan University of Medical Sciences, Isfahan, Iran; b Professor of Internal Medicine and Endocrinology, Isfahan Endocrine and Metabolism Research Center, Isfahan University of Medical Sciences, Isfahan, Iran; c Researcher, Isfahan Endocrine and Metabolism Research Center, Isfahan University of Medical Sciences, Isfahan, Iran

**Keywords:** Prediabetic States, Diabetes Mellitus, Type II, Oral Glucose Tolerance Test, Primary Prevention, Dyslipidemia, Risk Factor, Iran

## Abstract

**BACKGROUND:**

The aim of this study is to investigate the need for diabetes primary prevention program in isolated impaired fasting glucose (i-IFG) of the first degree relatives of type 2 diabetics.

**METHODS:**

In a cross sectional study, 793 individuals with prediabetes [543 with i-IFG and 250 with isolated impaired glucose tolerance (i-IGT)] who were the first degree relatives of type 2 diabetic patients, were enrolled. Isolated IFG was considered as fasting plasma glucose between 100-125 mg/dl and 2 hour plasma glucose < 140 mg/dl and isolated IGT as FPG < 100 mg/dl and 2 hour plasma glucose between 140-199 mg/dl during an overnight fasting 75 g oral glucose tolerance test. Mean of the age, weight, waist circumference, body mass index, systolic and diastolic blood pressure, plasma glucose, HbA1C, and lipid profile were compared between two groups (i-IFG and i-IGT). The prevalence of cardiometabolic risk factors (BMI ≥ 25 kg/m2, hypertension, cholesterol ≥ 200 mg/dl, LDL-C ≥ 100 mg/dl, HDL-C ≤ 40 mg/dl, and triglyceride ≥ 150 mg/dl) adjusted by age, sex and BMI were compared.

**RESULTS:**

The prevalence of cardiometabolic risk factors is higher in i-IFG group than i-IGT. The mean level of LDL-C is significantly higher in i-IFG than i-IGT group.

**CONCLUSIONS:**

First degree relatives of T2DM with isolated impaired fasting glucose should probably be included in the primary preventive program for diabetes. However, longitudinal cohort study is required to show high progression of i-IFG to T2DM.

Diabetes is a common chronic disease and a serious medical and social problem. The prevalence of type 2 diabetes mellitus (T2 DM) is rapidly increasing worldwide. It is estimated that by the year of 2025, the number of people with diabetes will increase to more than twice compared to the year of 2000.^1^

The prevalence of diabetes is reported to be 29% in the United Arab Emirates,^2^ and 16.1% in Oman.^3^ In Iran, this prevalence was 7.7% in people aged 25-65 years in 1993.^4^

Impaired glucose tolerance (IGT) and impaired fasting glucose (IFG) were initially recognized as conditions with increased risk of T2DM development.^5^ However, they are now considered to be the independent cardiovascular risk factors, specially, their combination (IFG + IGT), similar to obesity, hypertension and dyslipidemia.^6^–^8^

To estimate the seriousness of isolated IFG and IGT as cardiovascular risk factors and their progression to diabetes is very important in establishing a suitable diabetes prevention strategy.^1^

In this study, the mean of classical known cardiovascular risk factors and the main predictors of progression to T2DM was compared (cardiometabolic risk factors) in the first degree relatives of T2DM with isolated IFG and isolated IGT. This is a part of a cohort study to investigate the progression of these prediabetic states to diabetes.

## Methods

### 

#### Subjects

The present study was performed at the Isfahan Endocrine and Metabolism Research Center (IEMRC), Isfahan University of Medical Sciences, from 2004 to 2007. Patients with prediabetes (n = 793) were enrolled. They were selected by consecutive patients sampling from 35-55 years old people (n ≈ 3000) who were the first degree relatives of T2DM patients in Isfahan Diabetes Prevention Program Study.

All participants were interviewed for general demographic characteristics and current use of medications. The patients with T2DM, normal oral glucose tolerance, combined IFG + IGT, pregnant women and those who were taking corticosteroids were excluded from the study. Of 793 prediabetic patients, 543 had isolated IFG (187 males and 356 females) and 250 patients had isolated IGT (40 male and 210 female).

The study was approved by the IEMRC Medical Ethics Committee and all participants gave written consent. The research complied with the current version of the Declaration of Helsinki.

#### Anthropometric and Laboratory Measurements

All Participants underwent a 75 g oral glucose tolerance test after 10-12 hours of overnight fasting. Venous sampling was done after 0, 30, 60 and 120 minutes of glucose taking. The 2003 American Diabetes Association (ADA) criteria were used for definition of prediabetes.^9^ Isolated IFG was considered as fasting plasma glucose (FPG) between 100-125 mg/dl and 2 hour plasma glucose (2hPG) < 140 mg/dl. Isolated IGT was defined as FPG < 100 mg/dl and 2hPG between 140-199 mg/dl.

Anthropometric parameters including height, weight, and waist circumference (WC) were measured.

Body mass index (BMI) was calculated by weight in kilograms divided by the square of height in meters. Blood pressure (BP) was measured twice in a seating position after 5 minutes resting with a standard and calibrated mercury sphygmomanometer (ALPK2, Japan). Systolic blood pressure (SBP) ≥ 130 mmHg or diastolic blood pressure (DBP) ≥ 85 mmHg or taking medication for controlling high blood pressure was considered as having hypertension. Waist circumference was measured by standard method with a tape in a horizontal place around the abdomen at level of the iliac crest located on top of the iliac crest which did not compress the skin and was parallel to the floor in normal respiration.^10^

Plasma glucose was measured by GOD-PAP and HbA1C by ion-exchange chromatography methods. Total cholesterol and HDL-cholesterol (HDL-C) were measured by CHOD-PAP and triglyceride (TG) was done by GPO-PAP methods.

LDL-cholesterol (LDL-C) was calculated using friedewald formula when total triglyceride was less than 400 mg/dl.

#### Statistical Analysis

Statistical analysis of the data was performed using SPSS 13 for windows. Data were expressed as mean and standard deviation (SD). Independent t student test was used for the comparison of quantitative variables (age, waist circumference, BMI, blood glucose, HbA1C, lipid profile, systolic BP, and diastolic BP), between isolated IFG and isolated IGT groups.

Chi square test was used for the comparison of gender, as qualitative variable, between two groups.

The prevalence of cardiometabolic risk factors adjusted for age, sex and BMI, was compared between two groups, using multiple logistic regression. P values less than 0.05 were considered statistically significant.

## Results

This is a cross sectional study for comparison of demographic and cardiovascular risk factors and the main predictors of progression to T2DM (cardiometabolic risk factors) between 250 subjects with isolated IGT and 543 patients with isolated IFG who are the first degree relatives of type 2 diabetic patients.

General demographic characteristics, anthropometric measurements and systolic and diastolic blood pressure of participants are summarized in table 1.

**Table 1:** Mean and standard deviation of demographic, anthropometric and clinical characteristics of the first degree relatives of T2DM patients with isolated impaired glucose tolerance and isolated impaired fasting glucose

	i-IGT*	i-IFG**	P value
Age (years)	43.6 (7.2)	43.9 (6.6)	0.54
Gender:			
Male [n (%)]	40 (16%)	187 (34.4%)	0.001
Female [n (%)]	210 (84%)	356 (65.6%)	0.001
Weight (kg)	72.0 (10.6)	75.9 (11.9)	0.001
WC*** (cm):			
Male	96.2 (6.6)	95.5 (8.9)	0.6
Female	87.5 (9.1)	88.2 (8.9)	0.3
BMI (kg/m^2^)	29.1 (3.9)	29.3 (4.2)	0.4
Systolic BP(mmHg)	115 (15)	116 (16)	0.6
Diastolic BP(mmHg)	75 (13)	75 (12)	0.7

* i-IGT: Isolated impaired glucose tolerance

** i-IFG: Isolated impaired fasting glucose

*** WC: Waist circumference

Mean of body weight was statistically different in two groups (p < 0.01). However, this difference was not observed for BMI (p = 0.4). Neither a significant difference in waist circumference was observed between two groups.

Plasma levels of glucose during OGTT in i-IGT and i-IFG groups are presented in table 2. Results of standard OGTT after 0, 30, 60 and 120 minutes were statistically different in two groups (p < 0.01) (table 2).

**Table 2:** Oral glucose tolerance test results in the first degree relatives of type 2 diabetics with different glucose tolerance category

Time (min)	i-IGT* (mg/dl)	i-IFG** (mg/dl)	P value
0	91.1 (6.5)	107.2 (6.5)	0.001
30	148 (27.6)	156.1 (31.1)	0.001
60	173.1 (34.8)	154 (39.7)	0.001
120	158.4 (15.5)	106.6 (20.6)	0.001

* i-IGT: Isolated impaired glucose tolerance

** i-IFG: Isolated impaired fasting glucose

Table 3 presents the mean of plasma lipid concentrations and HbA1C in the two groups with different glucose tolerance category. There is only significant difference in LDL-C between two groups (p = 0.02).

**Table 3:** Plasma lipid profile and glycosylated hemoglobin in the first degree relatives of type 2 diabetics with different glucose tolerance category

	i-IGT*	i-IFG**	P value
Triglyceride (mg/dl)	172.7 (94.2)	167.7 (105.0)	0.5
Cholesterol (mg/dl)	196.5 (39.9)	201.5 (41.0)	0.5
HDL-c (mg/dl)	46.5 (12.4)	46.5 (13.4)	0.9
LDL-c (mg/dl)	116.7 (33.5)	123.2 (36.2)	0.02
HbA1C (%)	5.03 (0.74)	5.05 (0.74)	0.7

* i-IGT: Isolated impaired glucose tolerance

** i-IFG: Isolated impaired fasting glucose

The prevalence of cardiometabolic risk factors adjusted for age, sex and BMI and their comparison between two groups is shown in table 4. The prevalence of known cardiometabolic risk factors (BMI ≥ 25 kg/m^2^, hypertension, cholesterol ≥ 200 mg/dl, LDL-C ≥ 100 mg/dl, HDL-C ≤ 40 mg/dl, and triglyceride ≥ 150 mg/dl) was higher in i-IFG than i-IGT group in the first degree relatives of T2DM.

**Table 4:** Prevalence of cardiometabolic risk factors in the first degree relatives of T2DM patients with different glucose tolerance category

	Total	Female [n (%)]	Male [n (%)]	OR (CI 95%)
BMI ≥ 25 kg/m^2^:	682			
i-IFG*	464	310 (66.8)	154 (33.2)	1
i-IGT**	218	182 (83.5)	182 (83.5)	0.002 (0.000-0.267)
Hypertension:	213			
i-IFG	145	94 (64.8)	51(35.2)	1
i-IGT	68	55 (80.9)	13 (19.1)	0.43 (0.218-0.872)
Cholesterol ≥ 200 mg/dl:	366			
i-IFG	264	172 (65.2)	92 (34.8)	1
i-IGT	102	86 (84.3)	16 (15.7)	0.34 (0.193-0.628)
LDL-c ≥ 100 mg/dl:	529			
i-IFG	376	256 (68.1)	120 (31.9)	1
i-IGT	153	132 (86.3)	21 (13.7)	0.014 (0.000-0.702)
HDL-c ≤ 40 mg/dl:	238			
i-IFG	162	84 (51.9)	78 (48.1)	1
i-IGT	76	58 (76.3)	18 (23.7)	0.272 (0.109-0.681)
TG ≥ 150 mg/dl:	368			
i-IFG	248	150 (60.5)	98 (39.5)	1
i-IGT	120	92 (76.7)	28 (23.3)	0.035 (0.000-20.6)

* i-IFG: Isolated impaired fasting glucose

** i-IGT: Isolated impaired glucose tolerance

## Discussion

The results from this cross sectional study demonstrate that in the first-degree relatives of T2DM, prevalence of cardiometabolic risk factors adjusted by age, sex and BMI in i-IFG are higher in comparison to i-IGT group (Table 4).

Among these cardiometabolic risk factors, mean level of LDL-C is significantly higher in i-IFG than i-IGT group.

Higher BMI, weight gain, dyslipidemia, hypertension and elevated fasting plasma glucose are of the main predictors of progression to T2DM.^11^ The present findings show that i-IFG group has equal or higher prevalence of cardiometabolic risk factors than i-IGT group.

IFG and IGT have different pathophysiological mechanism. The main defects in subjects with IFG are the increased hepatic glucose output production and early insulin secretion dysfunction. Subjects with IGT have moderate to severe insulin resistance in level of muscles.^7^ IGT and IFG are risk factors of cardiovascular disease and progression to T2DM development. However, the degree of their influences on the cardiovascular disease and T2DM development is different.^7^,^12^,^13^

However, according to some previously done studies, IGT had stronger association with cardiovascular disease and T2DM development than IFG. The combination of IFG and IGT (IFG + IGT) had the strongest association in this regard.^7^,^13^,^14^ Therefore, primary prevention programs for T2DM were recommended in subjects with IGT or combined IFG + IGT.^7^,^13^ The above mentioned results are not in accordance with the present findings. It may be due to different population sampling. The participants in their research were general population; however, the present sampling was done on the first degree relatives of T2DM patients. Such people may have some metabolic and genetic characteristics which distinguish them from general population.

In some longitudinal studies, the cardiovascular disease (CVD) risks and progression to T2DM in future were higher in subjects with increased fasting plasma glucose.^15^ For example, in Framingham Offspring Free of CVD Study, women with FPG between 110 mg/dl and 125 mg/dl had higher risk for CVD than men.^16^ However, that study had not been performed on the first degree relatives of T2DM and in contrast to the present study, IGT subjects were not evaluated for CVD risk and T2DM.

In Australian Diabetes, Obesity and Lifestyle Study (AusDiab), 10428 volunteers from general population with 5.2 years follow up were evaluated.^17^ They suggested that all groups with abnormal glucose metabolism may require diabetes preventive program.

In Shaw et al study, 3542 participants were followed for 5 years.^8^ The risk of cardiovascular disease and development of diabetes in future increased with increasing fasting plasma glucose.

Findings of Henry et al study demonstrated that impaired fasting glucose in male general population with moderate systolic hypertension significantly increased cardiovascular mortality.^18^

In comparison to the above mentioned studies, the present study has some different characteristics. The sample of this study consists of people who were the first degree relatives of T2DM and only isolated IFG and isolated IGT were compared.

The most important strategy to decrease the prevalence of T2DM, is establishing the primary prevention program in those people who have the highest risk for progression to T2DM. As the first degree relatives of T2DM patients have significant risk of diabetes progression, according to the present findings, it is recommended that subjects with i-IFG in the first degree relatives of T2DM probably should have a primary preventive program.

To select the population of the study from the first degree relatives of T2DM is a strong point of the present study. However, this research has some limitations. One of the limitations is its cross sectional nature which cannot find a cause and effect relationship. Another limitation is that it was not a multicenter study.

## Conclusions

First degree relatives of T2DM with isolated impaired fasting glucose should probably be included in the primary preventive program for diabetes. However, longitudinal cohort studies are required to show high progression of i-IFG to T2DM.
